# Rosai‐Dorfman disease: A case report of asymptomatic isolated renal involvement

**DOI:** 10.1002/ccr3.4132

**Published:** 2021-07-10

**Authors:** Alireza Abrishami, Pardis Ziaeefar, Sara Ebrahimi, Nastaran Khalili, Akbar Nouralizadeh, Reza Farjad

**Affiliations:** ^1^ Department of Radiology Shahid Labbafinejad hospital Shahid Beheshti University of Medical Sciences Tehran Iran; ^2^ School of Medicine Shahid Beheshti University of Medical Sciences Tehran Iran; ^3^ Cancer Immunology Project (CIP) Universal Scientific Education and Research Network (USERN) Tehran Iran; ^4^ School of Medicine Tehran University of Medical Sciences Tehran Iran; ^5^ Urology and Nephrology Research Center Shahid Labbafinejad hospital Shahid Beheshti University of Medical Sciences Tehran Iran

**Keywords:** case report, histiocytosis, MRI, PET/CT, renal, Rosai‐Dorfman disease

## Abstract

A possible diagnosis of RDD should be kept in mind when encountering a patient with raised plasma creatinine levels and renal mass. Timely diagnosis and management of RDD will help prevent future kidney loss.

## Graphical abstract

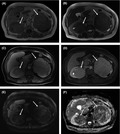

## 1 INTRODUCTION

Rosai‐Dorfman disease (RDD) is a rare nonmalignant histiocytosis disorder, commonly manifesting with massive painless cervical lymphadenopathy. Renal involvement develops in only four percent of patients with RDD. Generally, RDD is self‐limiting and has a good prognosis; however, in patients with renal involvement, mortality rate can be as high as 40%.

For the first time in 1969, Rosai and Dorfman described a new clinicopathologic entity of histiocytic proliferative disorders, which they named sinus histiocytosis with massive lymphadenopathy (SHML).^1^ SHML, also known as the Rosai‐Dorfman disease (RDD), is a non‐Langerhans cell histiocytosis disorder with unknown etiology that is histologically characterized by benign proliferation of S100‐positive histiocytes within the expanded sinus of lymph nodes and lymphatic vessels of visceral organs.^2^ This rare disease mainly affects children and younger adults but can also occur as late as the eighth decade.^3^ Evidence suggests that patients with a background of immunologic and autoimmune diseases are more susceptible to developing this disease.^4^, ^5^

Patients with RDD most commonly present with massive and bilateral painless enlargement of cervical lymph nodes, accompanied by nonspecific symptoms such as fever, night sweat, and weight loss. In approximately 40% of cases, RDD can also display extranodal involvement with the most common affected sites being the bone, skin, soft tissue, and upper respiratory tract.^6^ RDD is usually self‐limited and has an overall good prognosis; however, the extranodal involvement of vital organs, as well as the presence of immunological disorders, is associated with unfavorable outcomes and might require therapeutic interventions such as surgery or medical treatment.^5^, ^7^

Kidney involvement occurs in an estimated four percent of patients with extranodal RDD and is associated with a poor prognosis.^6^ Thus far, only two cases of isolated bilateral renal RDD have been reported, which had both been symptomatic at presentation.^8^, ^9^ Here, we report the third case of isolated bilateral renal RDD in a 67‐year‐old asymptomatic woman who presented with an elevated erythrocyte sedimentation rate (ESR) and increased serum creatinine level. In addition, we briefly review the radiologic findings of previously reported cases of renal RDD and compare them with other differential diagnoses.

## 2 CASE HISTORY/EXAMINATION

A 67‐year‐old woman presented with increased serum creatinine level and elevated erythrocyte sedimentation rate (ESR), which was detected during routine follow‐up. She did not complain of fever, weight loss, or any urinary symptoms. Except for an impalpable spleen, physical examination was otherwise normal. Her past medical history was positive for mild hypertension and antiphospholipid antibody syndrome (APAS) that had resulted in habitual abortion and thrombophlebitis of both lower extremities during pregnancy. The patient also reported a history of splenectomy following a motor vehicle accident. Approximately four years prior to this patient's further evaluation during a hospital admission, an abnormally high ESR (75 mm/h) was accidentally noticed during routine examinations, for which, considering her history of APAS, 10 mg oral prednisolone and 50 mg oral azathioprine were administered on daily basis. Although the patient had remained symptomless, a gradual rise in ESR (100 mm/h) was observed after one year from initiation of medical treatment. Further evaluation with computed tomography (CT) imaging had revealed a left renal mass that measured approximately 2‐3 cm in size; however, at that time, the patient had refused to undergo further assessment and continued receiving medical treatment.

### 2.1 Differential diagnosis, investigations, and treatment

**FIGURE 1:**
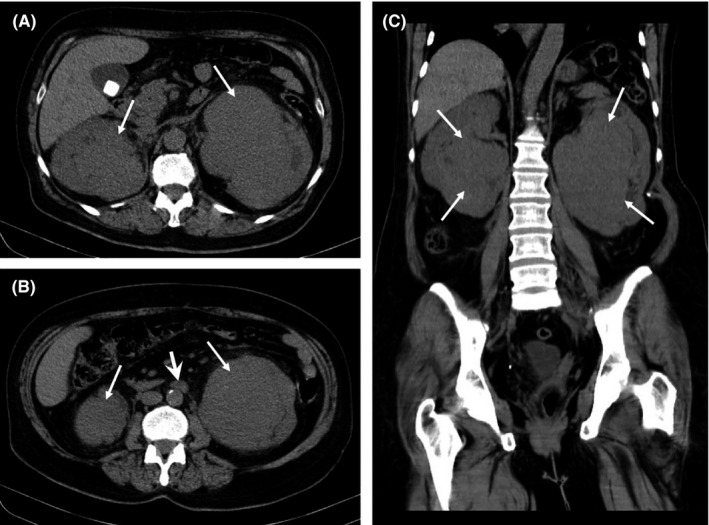
A‐C, Axial and reconstructed coronal images in a patient eventually diagnosed with Rosai‐Dorfman disease. A and C, Bilateral large masses are seen adjacent to the pelvicalyceal system (*thin arrows*) with extrarenal extension without calcification. B, Nonsignificant periaortic lymph node (*thick arrow*)

Upon hospital admission, routine laboratory tests and imaging were performed. Laboratory examination revealed elevated ESR (100 mm/h) and increased serum creatinine (1.6 mg/dL). Nonenhanced chest and abdominopelvic CT with reconstruction of volumes at 1mm slice thickness showed bilateral isodense renal pelvis masses without calcification and with a maximum diameter of 80 × 75 × 92 mm on the right and 90 × 102 × 126 mm on the left side. Multiple nonsignificant mediastinal and periaortic lymph nodes were also detected with a maximum short‐axis diameter (SAD) of 8 mm. No skeletal blastic or lytic lesions were noted (Figure 1).

**FIGURE 2:**
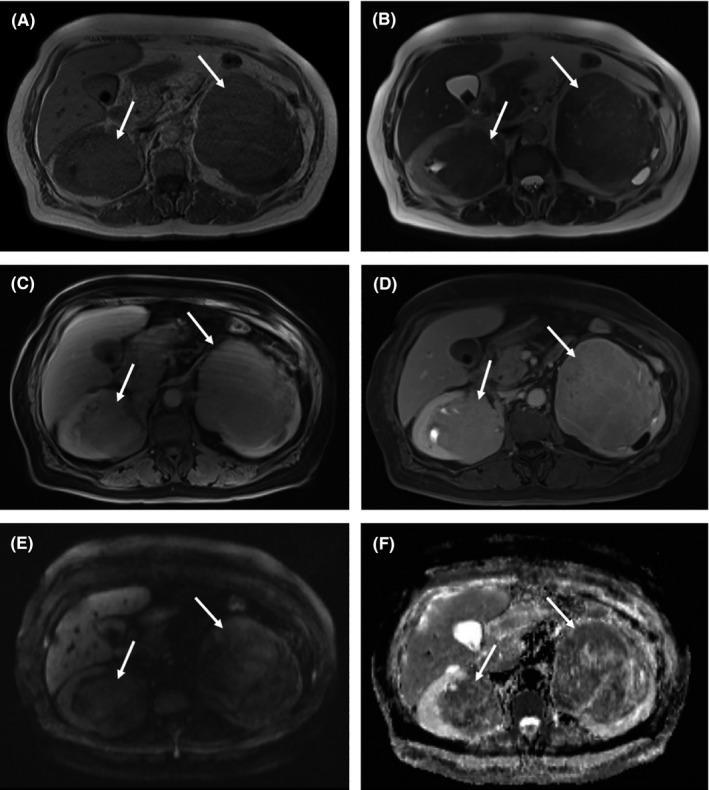
A‐F, Magnetic resonance imaging (MRI) of bilateral renal masses in a patient with Rosai‐Dorfman disease. A, Masses (*thin arrows*) have hypo‐to isointense signal on transverse T1‐weighted image and B, appear as mild heterogeneous hyperintense masses on transverse T2‐weighted image C, Masses have moderate enhancement on transverse gadoxetic acid‐enhanced nephrogenic phase image and D, sustain enhancement in the delayed phase. E, Mild hyperintense masses on diffusion‐weighted imaging obtained with a b‐value of 800 s/mm^2^ and F, hypointense masses on apparent diffusion coefficient map

For further evaluation of the renal lesions, abdominal magnetic resonance imaging (MRI) was carried out by a 1.5 Tesla scanner (Magnetom Avanto; Siemens Healthineers) using a sixteen‐channel phased array coil. MRI images included standard sequences such as T1‐weighted, T2‐weighted, diffusion‐weighted imaging (DWI) with b‐value of 50‐400‐800 s/mm^2^, and apparent diffusion coefficient (ADC) maps. In addition, contrast‐enhanced 3‐dimensional T1‐weighted gradient‐echo was performed in axial and coronal planes. The renal pelvis lesions displayed hypointense to isointense signals on T1‐weighted images and were isointense to hyperintense on T2‐weighted images with slight heterogeneity. On ADC maps, mild restricted diffusion with low signal intensity was exhibited. Masses had gradual enhancement without washout in the delayed phase. Bilateral hydronephrosis along with signs of parenchymal loss (predominantly in the left kidney) was also noted but neither perinephric nor subcapsular mass‐like lesions were seen. Other vital organs were preserved without any signs of infiltration, and no abnormal signal or enhancement was found in the periaortic lymph nodes (Figure 2). To reach a definite diagnosis, CT‐guided core needle biopsy was performed, which showed marked inflammatory cell infiltration with predominance of lymphocytes, plasma cells, and activated histiocytes without any evidence of granuloma formation; however, the histopathological findings were inconclusive.

Due to persistently raised creatinine, the patient subsequently became a candidate for surgery and underwent right partial nephrectomy and left radical nephrectomy with a small residual mass remaining due to technical difficulties of complete resection. Histopathologic examination of surgical specimens revealed diffuse histiocytic proliferation and plasma cell infiltration, which was in favor of a histiocytic proliferative disorder, and the subtle presence of emperipolesis raised the suspicion for RDD. Finally, immunohistochemical (IHC) assay stained positive for S100 protein and negative for CKAE1/AE3 and CD‐1a, confirming the diagnosis of RDD. Approximately 6%‐7% of the plasma cells also stained positive for IgG4.

### 2.2 Outcome and follow‐up

**FIGURE 3:**
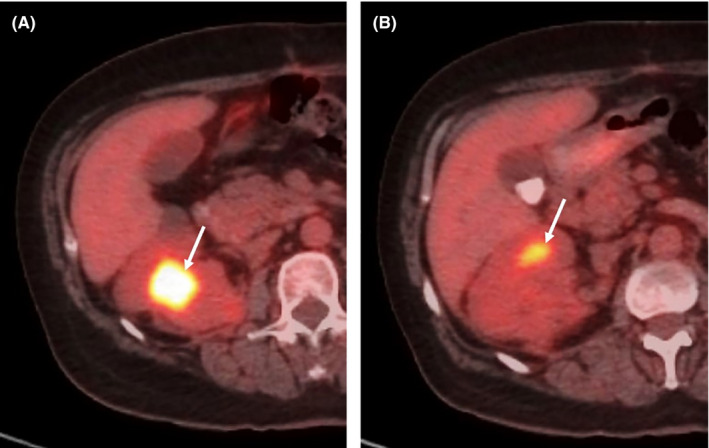
A and B, Follow‐up axial FDG PET/CT scan images of right renal mass and mediastinal lymph nodes in a patient with renal Rosai‐Dorfman disease. (A) The tumor in the right renal pelvicalyceal system (arrow) shows increased uptake (SUV_max_: 13.3) as well as (B) few renal cortical foci (arrow) with mild uptake (SUV_max_: 6.3)

On follow‐up, the patient developed resistant urinary tract infection two weeks after discharge, for which she was hospitalized and treated with intravenous meropenem. She also received daily nitrofurantoin as maintenance therapy for several months and continued to be followed by an oncologist with interval imaging. At three months postsurgery, renal sonography showed a simple cyst with a diameter of 19 mm and an echogenic mass lesion measuring 20 × 28 mm in the lower bridge and sinus of the right kidney (respectively) as well as mild hydronephrosis. Moreover, laboratory tests demonstrated normocytic anemia, elevated creatinine (1.4 mg/dL), and hematuria. Three months after the operation, at the oncologist's discretion, an FDG PET/CT scan (Discovery 690 VCT 64 Slicer, General Electric, USA) was performed for the patient that showed a hypermetabolic mass in the right kidney involving the pelvicalyceal system (SUV_max_: 13.3) and the cortex (SUV_max_: 6.3). Normal FDG activity was seen in the axial skeleton (Figure 3). As to the time of writing this report, the patient is alive and in good general condition. She is being monitored with imaging and ESR trend, and is receiving oral prednisolone (5 mg/ twice daily), as prescribed by her oncologist. The right renal mass lesion has decreased in size on follow‐up sonography, and her ESR level is currently 73mm/hour at five‐month postsurgery.

## 3 DISCUSSION

Here, we report the third case of isolated bilateral renal RDD without any evidence of significant adenopathy during diagnostic work‐up. RDD is a rare nonmalignant histiocytosis disorder with poorly understood pathogenesis. Frequent association with immunologic abnormalities suggests a probable immune‐mediated etiology.^5^ RDD most commonly manifests with massive painless cervical lymphadenopathy, occurring as a result of proliferation and aggregation of distinctive histiocytes within the expanded sinuses of lymph nodes. Nevertheless, it is not solely limited to the lymph nodes and lymphoid organs, as in 43% of cases, RDD manifests as extranodal involvement, either with or without associated lymphadenopathy.

Kidney involvement develops in only four percent of patients with RDD and is rarely isolated.^6^ In general, renal RDD is an indicator of poor prognosis, and due to complications of mass effect that compromises the function of the kidneys and adjacent organs, the mortality rate can be as high as 40%.^6^ Moreover, survivors usually suffer from chronic kidney disease.^10^ A history of autoimmune diseases, as in our case, also makes patients susceptible to a more severe disease course.^5^ This implies that early identification of patients with renal RDD, in particular those with a positive history of immunologic disorders, is of paramount importance.

Based on the site of involvement, RDD displays a wide range of nonspecific clinical and radiologic presentations, making its diagnosis challenging. The definite diagnosis of RDD must only be confirmed through histopathologic and IHC examination.^11^ In patients with indistinguishable radiological and clinical features, preoperative image‐guided biopsy could be worthwhile to avoid further invasive interventions; however, in some cases, as in our patient, needle biopsy is not adequate for drawing definite diagnosis and surgical biopsy is required. The histological hallmark of RDD is emperipolesis, in which lymphocytes, plasma cells, erythrocytes, and neutrophils are engulfed within histiocytes without being degraded.^1^ However, even this pathognomonic feature is less frequent in extranodal forms of RDD and might be difficult to identify.^5^ The most prominent IHC marker for distinguishing SHML histiocytes is the S100 protein. Other macrophage markers such as CD68 and CD163 also stain positive. However, the SHML histiocytes do not stain for CD1a and dendritic markers such as CD21, CD23, or CD35.^5^, ^6^ Also, as in our case, the absence of expression of markers such as CK AE1/AE3 helps differentiate renal RDD from carcinoma with epithelial origin such as ovarian or renal parenchymal tumors.^12^, ^13^

In patients with renal RDD, laboratory data range from normal to nonspecific findings such as leukocytosis, increased ESR, and hypergammaglobulinemia.^11^ Although imaging studies are also not highly discriminative, they will help narrow the diagnosis. Modalities such as renal ultrasound, abdominopelvic CT, and MRI, along with FDG PET/CT scan could be considered as beneficial tools for identification of the lesion, evaluation of the extent of disease, image‐guided biopsy, and even monitoring disease activity and response to therapy.^14^, ^15^ Due to the rarity of RDD, it is less likely for the urologist or radiologist to consider this diagnosis when observing an abnormal renal lesion on imaging; however, having a suspicion in mind might favorably change patient management and help prevent unnecessary nephrectomy.

The radiologic features of renal RDD include hilar masses, distinct nodules in the cortex, subcapsular infiltrative lesions, lobular irregularity, or kidneys with distorted calyces. Occasionally, large para‐aortic lymph nodes might also be noted.^4^, ^16^ Nevertheless, imaging findings of renal RDD may mimic that of important differential diagnoses such as Erdheim‐Chester disease, renal cell carcinoma, lymphoma, leukemia, multiple myeloma, or metastasis from melanoma or lung cancer ^17^; thus, it is essential to exclude the possibility of these potentially life‐threatening diseases. Table 1 compares the common imaging features of important differential diagnoses of RDD. In the case presented here, bilateral isodense renal pelvis masses without calcification were seen on unenhanced CT. MRI revealed hypo‐to isointense masses on T1‐weighted images and isointense to hyperintense signals on T2‐weighted images with slight heterogeneity. ADC maps were suggestive of mild restricted diffusion, and delayed phase imaging showed gradual enhancement of the masses without washout. In 1999, Bain and colleagues reported the first case of bilateral renal RDD. They noted heterogeneous hypoechoic masses with calcification foci on ultrasound and noncystic masses with soft tissue attenuation on unenhanced CT. They also performed contrast‐enhanced CT, which showed less homogeneous enhancement as compared with the renal parenchyma.^18^ Another study in 2001, describing three cases with RDD and renal involvement, showed that RDD manifests as infiltrative renal hilar masses and subcapsular infiltration on contrast‐enhanced CT.^19^ Gumeler and colleagues reported infiltrative renal hilar mass with ill‐defined borders as a characteristic feature of renal RDD and suggested hypo‐attenuated renal cortical nodules and subcapsular infiltration as other important imaging findings on CT. In the case described in their study, T1W and T2W imaging showed low signal intensity with mild enhancement on postcontrast images.^14^ In 2019, Kmetz et al reported the most recent case of bilateral renal RDD in a 60‐year‐old Caucasian man with a history of anemia and thrombocytopenia of unknown etiology. Subsequent to identifying a left upper quadrant mass on palpation, CT scan was performed for the patient, revealing bilateral soft tissue masses in the perinephric space, measuring up to 22 cm on the left side.^9^

**TABLE 1:** Common imaging features of important differential diagnoses of RDD

	Ultrasound	Computed Tomography	Magnetic Resonance Imaging	FDG PET/CT
Renal cell carcinoma	Solid to partially cystic mass that can be hyper‐, iso‐, or hypoechogenic compared with the normal renal parenchyma. The pseudocapsule of the tumor is sometimes seen as a hypoechoic halo sign.^23^	Lesions demonstrate soft tissue attenuation on nonenhanced CT. On contrast‐enhanced CT, they are usually enhanced less than the normal cortex. Smaller lesions display homogeneous enhancement, whereas larger lesions have irregular enhancement due to areas of necrosis. Degrees of calcification are seen in about 30% of cases.^24^	Lesions are heterogeneous on T1W. Clear cell RCC is hyperintense and papillary RCC is hypointense on T2W.^25^ A hypointense rim between the tumor and the normal renal parenchyma suggests tumor pseudocapsule.^26^	Lesions usually demonstrate mild FDG avidity.^27^
Renal Lymphoma	Single or multiple hypoechoic masses located within renal parenchyma with scarce internal vascularity. Varying degrees of hydronephrosis might also be seen.^28^	Wide range of findings including multiple poorly enhanced masses with significant retroperitoneal lymphadenopathy (most common), single homogeneous and hypodense mass without cystic change, retroperitoneal nodal mass invasion with or without hydronephrosis, diffuse infiltration without evidence of obvious mass, perirenal mass, or nodule. Atypical features include calcification, hemorrhage, necrosis, cystic change, or heterogeneous lesions ^27^, ^28^, ^29^	T1W shows hypointense signal and T2W exhibits iso‐ to hyperintense signal compared with normal parenchyma. Contrast‐enhanced T1 is indicative of poor and delayed (in some cases) enhancement.^28^	FDG uptake by the lymphoma lesions was much higher than the FDG uptake by the renal cell carcinomas.^30^
Erdheim‐Chester disease	Ultrasound demonstrates retroperitoneal and perirenal infiltration.^31^ Stenosis of renal arteries and decreased/ absent flow might be seen on duplex ultrasound due to perivascular fibrosis.^32^	Symmetric homogeneous hypo‐enhanced perinephric soft tissue that encases the kidneys, known as “hairy kidney” appearance. Hydronephrosis, calyceal dilatation, and bilateral ureteral encasement may also be seen in some cases.^2^	T1 and T2 imaging show perinephric soft tissue with isointense signal relative to muscle and mild enhancement after IV contrast.^27^	FDG PET/ CT shows increased uptake in the involved sites )due to increased glucose metabolism by histiocytes) ^27^
Multiple myeloma		Multiple enhancing perinephric nodules and masses (most common); focal renal masses can also be observed.^27^		Intense FDG uptake by the masses.^27^
Metastatic Lesion	Multiple metastases usually appear as small, poorly marginated, hypoechoic masses.^33^	Multiple, small, and bilateral lesions predominantly confined to the renal parenchyma. At CT and MR imaging, the contrast enhancement characteristics vary according to the site of the primary tumor.^27^	Multiple, small, and bilateral lesions predominantly confined to the renal parenchyma. Signal intensity depends on the primary site.^27^	Increased FDG uptake; greater than that of the adjacent renal parenchyma.^34^

For the first time in 2004, Yu et al demonstrated moderately increased FDG accumulation in mediastinal lymph nodes affected by RDD, mainly due to excess radioglucose metabolism of the proliferating histiocytes and other inflammatory cells.^20^ After that, several studies investigated the usefulness of 18F‐FDG PET/CT for evaluating nodal and extranodal RDD. In general, the findings of 18F‐FDG PET/CT are nonspecific for RDD and histological examination is still needed to establish the diagnosis; however, this modality is valuable for assessment of disease burden and predicting prognosis. Furthermore, it could be advantageous for monitoring response to therapy and detecting foci of disease progression during follow‐up.^15^, ^21^, ^22^ This will consequently facilitate decision‐making and treatment planning for patients with RDD.

Conclusively, as demonstrated in this case, when encountering a renal mass on imaging with an accompanying renal dysfunction, the clinician should be aware to keep the diagnosis of RDD in mind and consider further evaluation as soon as possible, even in a symptomless patient. This will help to favorably change the patient's management and prevent unnecessary nephrectomy. Furthermore, performing FDG PET/CT scan on follow‐up can be beneficial for evaluation of disease extension, monitoring of disease, and planning future treatment strategies in patients with renal RDD.

## CONFLICT OF INTEREST

None declared.

## AUTHOR CONTRIBUTIONS

AA: took part in conceptualization and study design, methodology, data curation, reviewing and editing, and supervision of the study; PZ: took part in data curation and literature search and writing the original draft. SE: participated in data curation and literature search. NK: participated in literature search and writing the original draft. AN: performed the surgery and reviewing and editing of the study. RF: took part in reviewing and editing and supervision of the study. All authors read and approved the final manuscript.

## ETHICS APPROVAL AND CONSENT TO PARTICIPATE

This study was approved by the ethics committee of Shahid Beheshti University of Medical Sciences and written informed consent was obtained from the patient.

## CONSENT FOR PUBLICATION

Written informed consent was obtained from the patient for permission of publication.

## Data Availability

The datasets used and/or analyzed during the current study are available from the corresponding author on reasonable request.
